# MiR-1a-3p Inhibits Apoptosis in Fluoride-exposed LS8 Cells by Targeting *Map3k1*

**DOI:** 10.1007/s12011-023-03869-9

**Published:** 2023-10-02

**Authors:** Ting Chen, Yu Gu, Guo-Hui Bai, Xia Liu, Bin Chen, Qin Fan, Jian-Guo Liu, Yuan Tian

**Affiliations:** 1 Key Laboratory of Oral Disease Research, School of Stomatology, Zunyi Medical University, Zunyi, 563000 China; 2 Loudi Central Hospital, Loudi, China

**Keywords:** miRNA, Apoptosis, Dental fluorosis, MAPK pathway, miR-1a-3p

## Abstract

Dental fluorosis is a common chemical disease. It is currently unclear how fluorosis occurs at the molecular level. We used miRNA-seq to look at the differences between miRNAs in the cell line of ameloblasts LS8 that had been treated with 3.2 mmol/L NaF. We also performed gene ontology (GO) and Kyoto Encyclopedia of Genes and Genomes (KEGG) pathway enrichment analyses. miR-1a-3p levels were significantly lower in mouse LS8 cells treated with 3.2 mmol/L NaF, and miR-1a-3p-targeted genes were significantly enriched in the MAPK pathway. LS8 cells were divided into four groups: control, NaF, NaF+miR-1a-3p mimics, and NaF+miR-1a-3p mimics normal control groups. Cellular morphology was observed by an inverted microscope, and the proliferation activity of LS8 cells was assessed by Cell Counting Kit-8 (CCK-8). Using the real-time quantitative polymerase chain reaction (RT-qPCR), transcription levels of miR-1a-3p and Map3k1 were detected. The expressions of Bax, Bcl-2, Map3k1, p38MAPK, ERK1/2, p-p38MAPK, and p-ERK1/2 were measured by Western blot. After bioinformatics analysis, we used a luciferase reporter assay (LRA) to validate the target of miR-1a-3p, showing that miR-1a-3p could inhibit apoptosis while increasing proliferation in fluoride-exposed LS8 cells. Generally, miR-1a-3p might directly inhibit Map3k1, reduce MAPK signal pathway activation, and promote phosphorylation. Thus, our findings revealed that the interaction of miR-1a-3p with its target gene *Map3k1* and MAPK signal pathway might decrease the apoptosis of LS8 cells treated with 3.2 mmol/L NaF.

## Introduction

Endemic fluorosis is a chemical disease that affects people all over the world. It is also one of the most serious diseases that naturally occur in China. Dental fluorosis is a precursor and specific indicator of chronic fluorosis in the oral cavity and has a significant impact around the world. When dental fluorosis develops, it not only changes how a person looks but can also affect their mental health in different ways. According to the results of the fourth oral epidemiological survey, 13.4% of 12-year-olds in China have dental fluorosis [1]. It is currently unclear why tooth fluorosis occurs, and its molecular mechanisms are not completely clear. Since the abnormal posttranscriptional cascades causing bone and tooth deformities are unknown, there is no prevention and treatment for dental fluorosis. LS8 cells are the key cells in the development of enamel. Many studies showed that excessive fluoride can damage LS8 cells and cause dental fluorosis [2, 3]. The MAPK pathway is an important pathway that affects the formation of enamel, matrix secretion, and cusp formation [4]. Odontoblast differentiation is also affected by the MAPK signal pathway [5]. Fluoride can mediate the expressions of downstream genes through the MAPK signal pathway to participate in dental fluorosis development [6–8].

microRNA (miRNA) is an endogenous (18–25 nt) nonprotein coding RNA molecule that regulates posttranscriptional gene expression by pairing with 3'UTR of the target mRNA. Many pathophysiological processes are regulated by miRNAs [9]. Studies demonstrated that miRNAs play an important role in enamel development by regulating epithelial cell differentiation, enamel mineralization, and extracellular matrix receptor interaction. In recent years, epigenetic studies on fluorosis have emerged. Some studies reported that miRNAs affect epigenetic modification by regulating target genes, and miRNAs and fluoride are closely related. Excessive fluoride exposure may significantly disrupt the expression pattern of miRNA [10–12]. Thus far, the important role of miRNA in fluorosis and tooth development has been widely recognized. Therefore, our team thought that ameloblast apoptosis caused by excessive fluoride might involve changes in miRNA expression. The experimental results showed that excessive sodium fluoride promoted apoptosis of LS8 cells and inhibited their proliferation, and the effect increased with concentration and time. In this study, we analyzed previous sequencing data to find differentially expressed miRNAs of LS8 cells undergoing apoptosis and treated with 3.2 mmol/L NaF and explore the mechanism of regulating fluoride-stained apoptosis of LS8 cells.

## Materials and Methods

### Cell Source

The cell line of mouse ameloblastic LS8 cells was donated by Professor Malcolme Snead of the University of Southern California and Professor Xiaohong Duan of the fourth military Medical University.

### Bioinformatics Analysis of Differentially Expressed miRNAs in LS8 Cells Treated with 3.2 mmol/L NaF for 24 h

Bioinformatics analysis of LS8 cells treated with 3.2 mmol/L NaF for 24 h showed that some miRNAs were expressed differently than others. This information came from previous research on miRNA sequencing. Through screening, we obtained differentially expressed miRNAs of LS8 cells treated with high concentrations of fluoride. There were two groups: 0 mmol/L and 3.2 mmol/L groups. The screening criteria for differential miRNAs were |log_2_FC| ≥1.5 and Q value <0.05. Target genes were predicted and screened by the “multimiR package” in R language (sum ≥ 4). Using the R language “clusterprofiler 4.0 package,” the gene ontology (GO) and Kyoto Encyclopedia of Genes and Genomes (KEGG) pathway enrichment analyses of each miRNA predicted differential target gene were performed.

### Cell Culture

LS8 cells were cultured using D-MEM (Livning, China) high-glucose medium with 10% fetal bovine (Livning, China) serum and 1% penicillin-streptomycin (Livning, China) mixture in a 5% CO_2_ incubator at 37 °C. In the same way, the cells were divided into two groups: 0 mmol/L and 3.2 mmol/L groups. LS8 cells were inoculated into a 10-mm-diameter Petri dish at a density of 1 × 10^4^/mL. NaF was not added in the 0 mmol/L group, and other culture conditions were the same as described above. In 24 h, the morphology of cells was observed under an inverted microscope and photographed.

### Transfection

miR-1a-3p mimics and miR-1a-3p mimics normal control (NC) were purchased from Ribobio (Guangzhou, China). LS8 cells were inoculated into 6-well plates at a density of 1 × 10^4^/mL. The cells were divided into control, NaF, NaF+miR-1a-3p mimics, and NaF+mimics NC groups. When the cell growth density reached 30–50%, the cells were transfected according to the instructions in the transfection kit. After 24 h of growing in the incubator, the cells were treated with 3.2 mmol/L NaF in a medium for 24 h.

### RNA Isolation and qPCR

The Total RNA Extraction Kit (Solarbio, China) was used to perform RNA extractions. For miRNA research, RNAs were reversed into cDNA by miRNA First Strand cDNA Synthesis (Sangon, China). Quantitative polymerase chain reaction (qPCR) was performed on the detection system with Universal SYBR® Green Master Mix (Roche Light Cycler 96). β-actin and U6 were used as internal references. The results of cell lines were computed by the 2^−ΔΔCt^ method. Primer sequences are listed in Table 1.

**Table 1:** Primer sequences used for RT-qPCR

Gene or miRNA	Sequence (5′to 3′)
mmu-miR-1a-3p	CGCACGCGTGGAATGTAAAGAAGTATG
U6 (forward)	CTCGCTTCGGCAGCACA
U6 (reverse)	AACGCTTCACGAATTTGCGT
Map3k1 (forward)	TTATCGGGCCTCAGAACTGC
Map3k1 (reverse)	ATGGTGTTACGAGACGGAGC
Ovine β-actin (forward)	CCAAAGCCAACCGTGAGAA
Ovine β-actin (reverse)	AGAGGCGTACAGGGACAGCA

### Cell Proliferation

LS8 cells of each group were inoculated at a density of 1 × 10^4^ cells per well on 96-well plates. When the cell growth density was about 30–50%, the cells were transfected for 24 h and then treated with 3.2 mmol/L NaF for 24 h. The CCK-8 detection kit instructions were followed, and the absorbance of each well was measured using the enzyme-linked immunosorbent assay at 450 nm.

### Western Blot

Bax, Bcl-2, and β-actin antibodies were purchased from Meilunbio (Dalian, China). p38MAPK, p-p38 MAPK, ERK1/2, and p-ERK1/2 antibodies were purchased from Ambart. The respective proteins were then exposed and analyzed with ImageJ software.

### Luciferase Reporter Assay (LRA)

Wild-type and mutant plasmids were constructed by Ribobio. m-Map3k1-WT and m-Map3k1-MUT were the wild-type and mutant 3'UTR-recombinant plasmids, respectively. The plasmids were co-transferred into LS8 cells. After 48 h of induction, the double luciferase report kit was used to measure fluorescence and normalize the final intensity.

### Statistical Analysis

All measurement data were expressed as mean ± standard error of the mean. Difference between groups was analyzed with the t-test and one-way analysis of variance for multiple comparisons. *P <* 0.05 indicated a statistically significant difference.

## Results

### Bioinformatics Analysis of miRNA Differential Expressions in LS8 Cells Treated with 3.2 mmol/L NaF

**Fig. 1:**
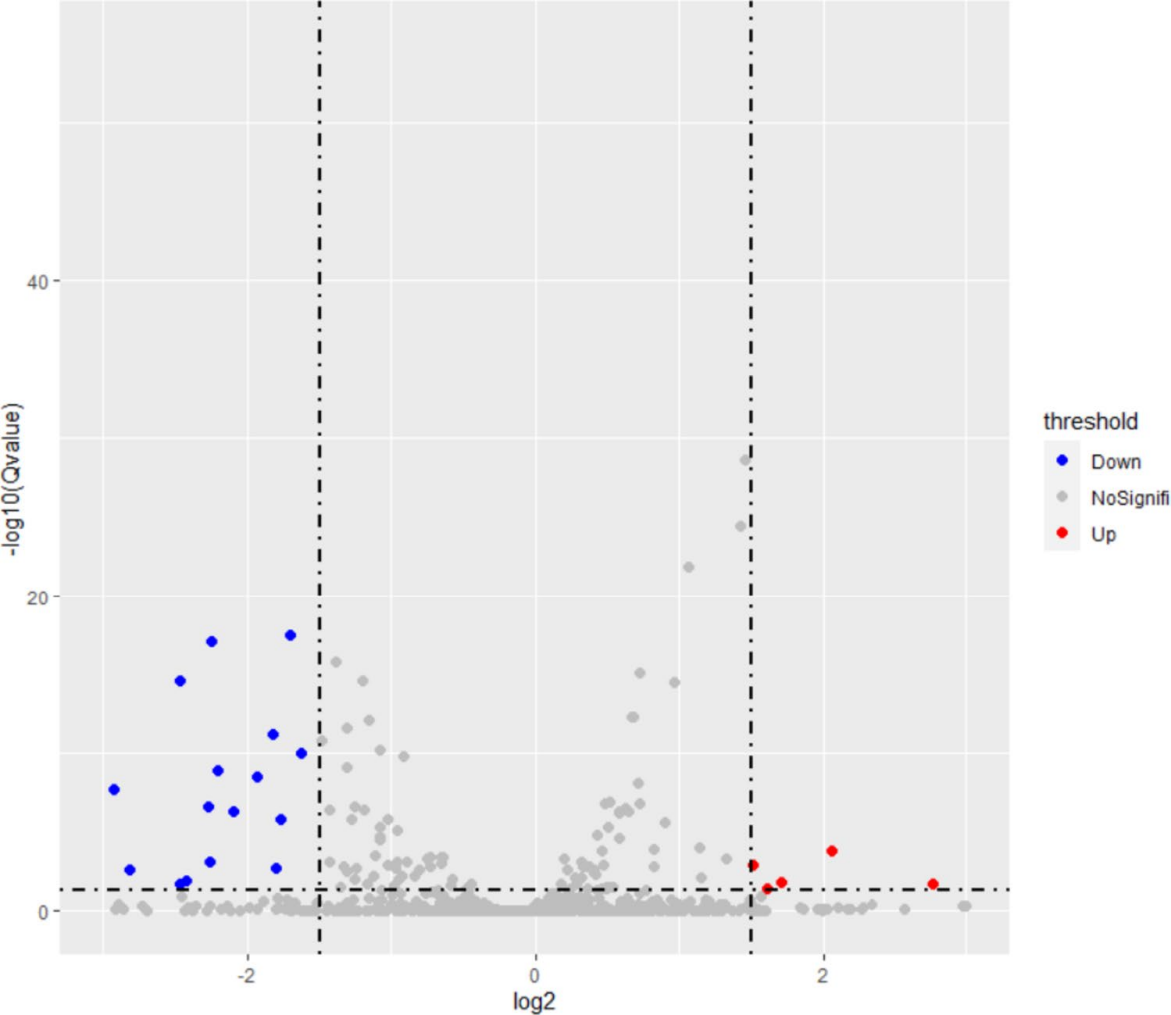
Differential miRNAs volcano map (R ggplot2 package). The red dots represent differential miRNAs that are up-regulated, while the blue dots represent down-regulated ones

Our previously miRNA sequencing data from BGI (Beijing Genomics Institute) and differentially expressed miRNAs of 3.2 mmol/L and 0 mmol/L NaF treatment groups were analyzed regarding fluoride-induced enamel cell apoptosis miRNA. A total of 28 differential miRNAs were screened from 1177 miRNAs (Fig. 1). Table 2 shows that mmu-miR-1a-3p was significantly down-regulated.

**Table 2:** The expression of apoptosis-related differential miRNAs in LS8 cells treated with 3.2 mmol/L NaF for 24 h

miRNA	Control Expression	Treat Expression	Expression
mmu-miR-1938	28.46	40.14	↑
mmu-miR-7b-5p	885.14	1518.849	↑
mmu-miR-216a-5p	15.477	69.249	↑
mmu-miR-196b-5p	15.171	18.905	↑
mmu-miR-204-5p	105.865	149.471	↑
mmu-miR-219a-5p	0.732	3.81	↑
mmu-miR-615-5p	14.023	5.198	↓
mmu-miR-298-5p	0.0711	0.1314	↓
mmu-miR-1224-5p	18.358	14.408	↓
mmu-miR-1a-3p	9.014	0.47	↓
mmu-miR-1983	50.635	72.448	↓
mmu-miR-212-3p	2.901	6.401	↓
mmu-miR-216a-5p	1.267	3.623	↓
mmu-miR-218-1-3p	2.056	4.559	↓
mmu-miR-219a-5p	0.732	3.053	↓
mmu-miR-210-5p	10.474	2.734	↓
mmu-miR-214-3p	733.587	338.156	↓
mmu-miR-23b-5p	15.039	3.149	↓
mmu-miR-26a-2-3p	58.785	6.758	↓
mmu-miR-26b-3p	31.478	12.665	↓
mmu-miR-297a-5p	525.08	334.802	↓
mmu-miR-298-3p	14.023	5.198	↓
mmu-miR-326-3p	20.984	10.689	↓
mmu-miR-351-3p	9.072	2.12	↓
mmu-miR-365-1-5p	2.902	0.603	↓

### GO and KEGG Enrichment Analyses

**Fig. 2:**
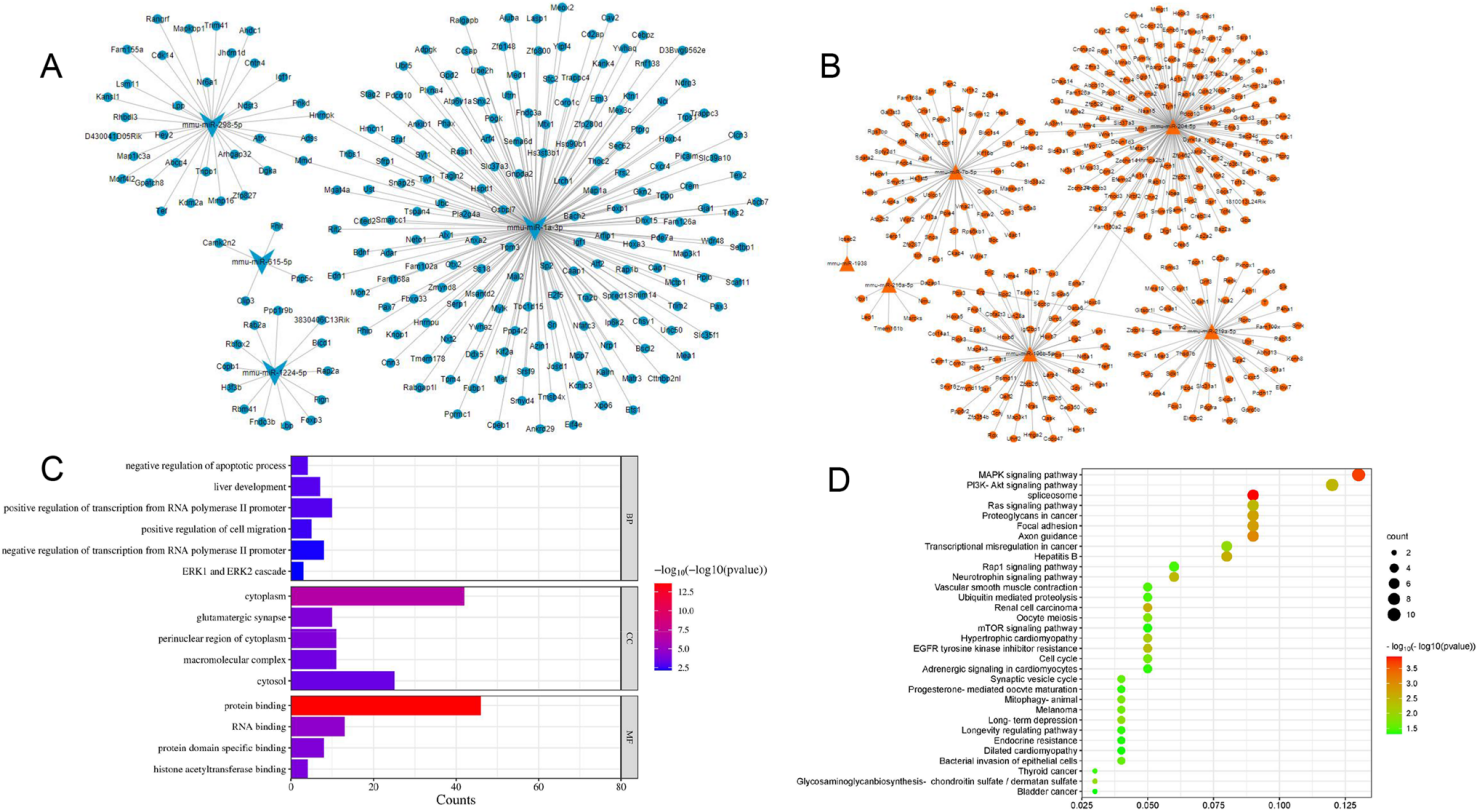
Partially down-regulated (**A**) and up-regulated (**B**) differential miRNAs’ target genes prediction map. MiR-1a-3p-targeted genes GO functional enrichment map (**C**) and KGEE pathway enrichment map (**D**)

GO and KEGG pathway enrichment analyses were performed to analyze differential miRNAs from target genes. The target genes of miR-1a-3p were significantly enriched in biological processes such as apoptosis, gene expression, protein synthesis, and RNA synthesis. Further pathway enrichment analysis of predicted target genes showed that differential miRNAs were enriched to 32 pathways, of which the MAPK pathway enrichment was the most prominent (Fig. 2).

### Morphological Changes and miR-1a-3p Expression of Fluoride-stained LS8 Cells

**Fig. 3:**
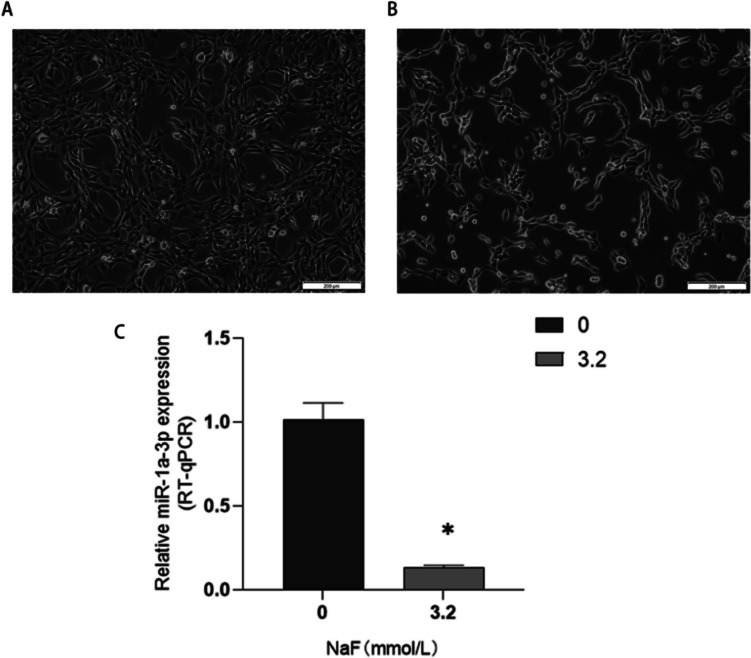
Morphology of LS8 cells in normal group (**A**) and NaF group (**B**). The expression of miR-1a-3p in LS8 cells decreased significantly (**C**). *Compared with 0 mmol/L group, *P* < 0.05

Normal LS8 cells were fusiform or triangular and in normal shapes with tight connections (Fig. 3A). When treated with 3.2 mmol/L NaF for 24 h, LS8 cells shrank, the number of apoptotic cells increased, and cells were successfully stained with fluoride (Fig. 3B). Reverse-transcription (RT)-qPCR showed that the expression of miR-1a-3p in 3.2 mmol/L NaF group was significantly lower than that of the 0 mmol/L group (*P* < 0.05). The results were consistent with sequencing data (Fig. 3C).

### Effects of miR-1a-3p on Morphology, Proliferation, and Apoptosis of Fluoride-exposed LS8 cells

**Fig. 4:**
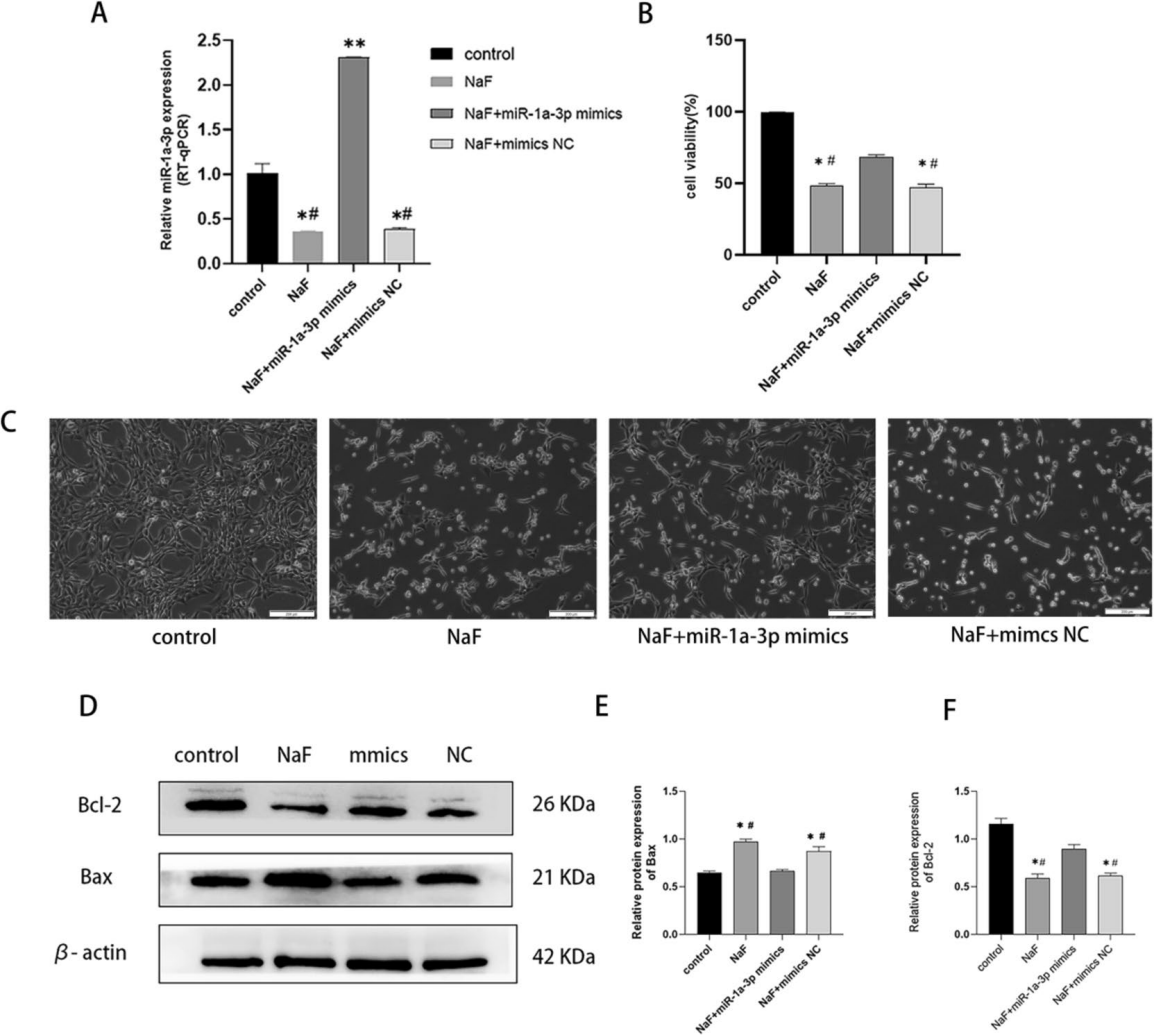
The expressions of miR-1a-3p were significantly increased after miR-1a-3p mimics transfection into LS8 cells (**A**). MiR-1a-3p promoted the proliferation of fluoride-stained LS8 cells (**B**), induced the growth of fluoride-stained LS8 cells and slightly changed their morphology (**C**), and inhibited the expression of Bax and increased the the expression of Bcl-2 (**D**,** E**,** F**). *Compared with the control group, *P* < 0.05; **compared with the control group, *P* < 0.01; #compared with the NaF+miR-1a-3p mimics group, *P* < 0.05

After the transfection of miR-1a-3p mimics into LS8 cells, the expression of miR-1a-3p increased significantly (Fig. 4A). Compared with the control group, the number of floating cells in NaF, NaF+miR-1a-3p mimics, and NaF+miR-1a-3p mimics NC groups significantly increased, and these cells shrank to different degrees after fluoride exposure. After miR-1a-3p mimics transfection, LS8 cells proliferated well but changed their form from long fusiform to short fusiform. The cells could still form a grid and had no morphological changes (Fig. 4C). After fluoride treatment, NaF, NaF+miR-1a-3p mimics, and NaF+miR-1a-3p mimics NC groups had significantly decreased cell proliferation activity than the control group, while the cell proliferation activity of the NaF+miR-1a-3p mimics group was significantly higher than that of the NaF group. NaF+mimics NC and control groups had similar cell viability (Fig. 4B). The expression of Bax increased, that of Bcl-2 decreased, and the ratio of Bax/Bcl-2 increased in the NaF group compared to the control group, but there was no significant difference between NaF+miR-1a-3p mimics NC and NaF groups. Compared with the NaF group, the overexpression of miR-1a-3p inhibited the apoptosis-inducing effect of excessive fluoride on LS8 by decreasing the expression of proteins Bax and Bax/Bcl-2 ratio in the NaF+miR-1a-3p mimics group (Fig. 4D, E, F). Thus, compared with the NaF group, the overexpression of miR-1a-3p can inhibit apoptosis and promote the proliferation of fluoride-stained LS8.

### miR-1a-3p Targeting Map3k1 Regulates MAPK Signal Pathway

**Fig. 5:**
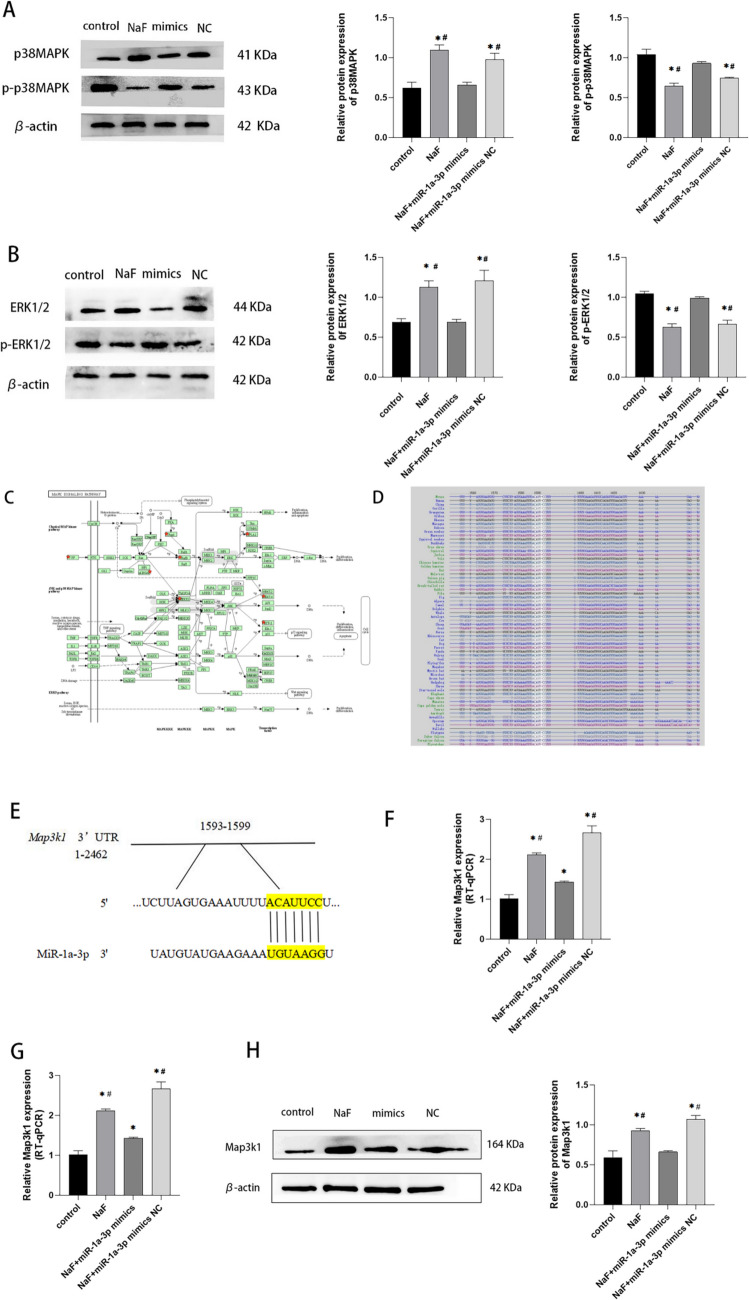
MiR-1a-3p targeting Map3k1 regulates MAPK signal pathway. MiR-1a-3p inhibits the expression of p38MAPK and ERK1/2 but promotes the expression of p-p38MAPK and p-ERK1/2 (**A**, **B**). Map3k1 located at the central node of MAPK pathway (**C**). The sequence of *Map3k1*3' UTR seed region among different species is highly conserved (**D**). Map3k1 is the direct target of miR-1a-3p (**E**), and miR-1a-3p can bind to the highly conserved target of Map3k13' UTR region (1593-1599 5’-ACAUUCC-3’) (**F**). The expression of Map3k1 in fluorine-exposed LS8 cells is inhibited by Gregory Homerimi miR-1a-3p (**G**, **H**). *Compared with the control group, *P* < 0.05; #compared with the NaF+miR-1a-3p mimics group, *P* < 0.05

**Fig. 6:**
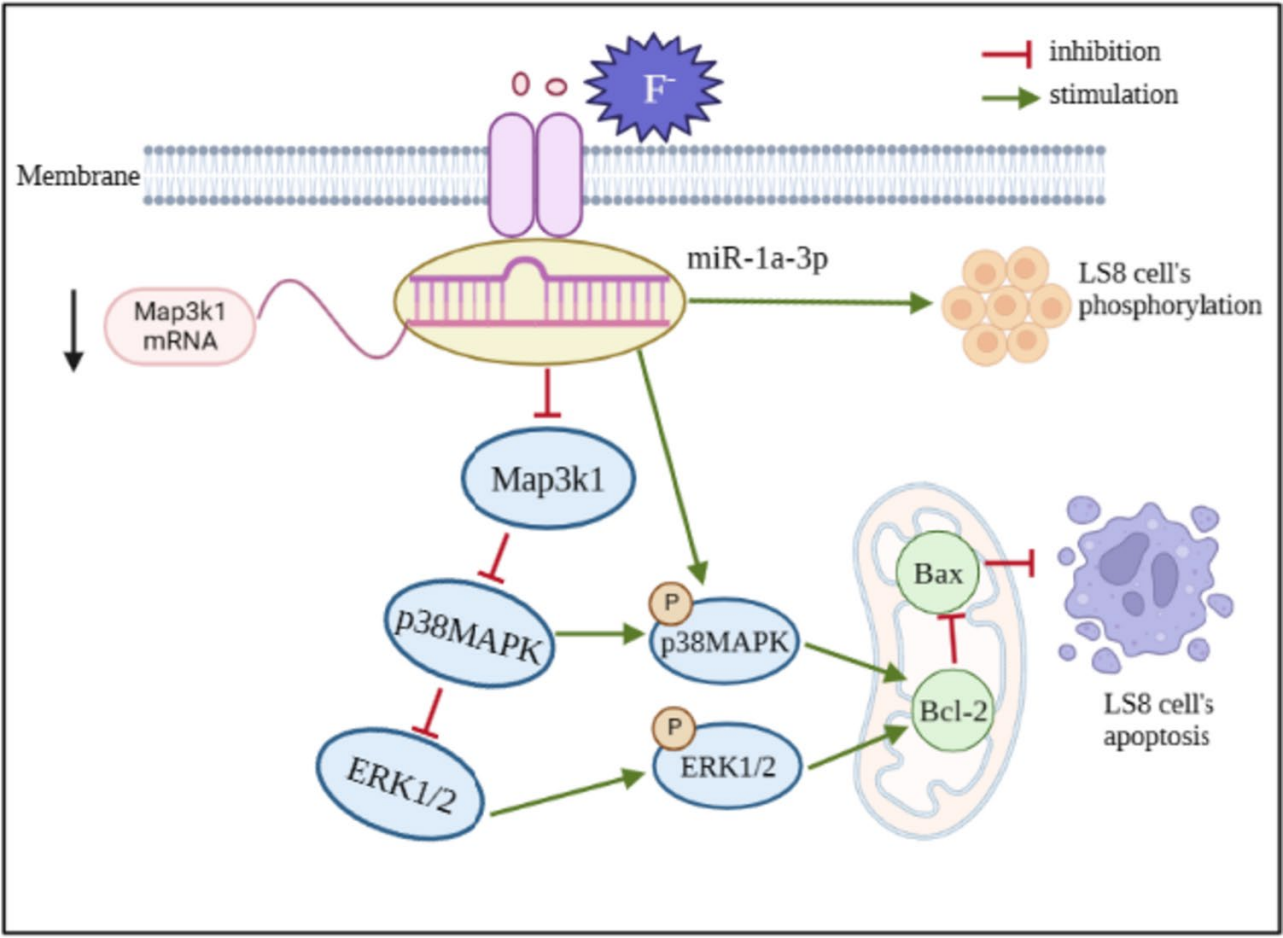
MiR-1a-3p inhibits apoptosis in fluoride-exposed LS8 cells by targeting *Map3k1 via* MAPK signal pathway

Western blot was used to detect the expression of MAPK pathway-related proteins p38MAPK, ERK1/2, and their phosphorylated forms in each group (Fig. 5A,B). The results showed that the NaF group had a higher level of MAPK signal pathway proteins p38MAPK and ERK1/2 but lower p-p38MAPK and p-ERK1/2 expressions than the control group. We further explored target genes of the MAPK signal pathway that are regulated by miR-1a-3p. The MAPK signal pathway had nine differential target genes controlled by miR-1a-3p (Table 3). Target gene *Map3k1* of miR-1a-3p is located in the central node of the MAPK pathway (Fig. 5C), which is involved in cell proliferation and apoptosis. Therefore, we picked it as the object of study. Through the target gene prediction analysis, a predicted binding site was found (Fig. 5E) in the seed region sequence of miR-1a-3p and 3'UTR of *Map3k1* (Fig. 5E), and *Map3k1* 3'UTR seed region sequence was highly conserved across species (Fig. 5D). The fluorescence activity of m-Map3k1-WT and m-Map3k1-MUT was analyzed after cotransfection with mmu-miR-1a-3p mimics and mmu-miR-1a-3p mimics NC into LS8 cells. LRA demonstrated that m-Map3k1-WT had lower fluorescence expression than m-Map3k1-MUT (*P* < 0.01). Then, the mRNA and protein expressions of Map3k1 were detected in each group. The results showed that the overexpression of miR-1a-3p inhibited transcriptional and translational levels of Map3k1 in fluoride-stained LS8 (Fig. 5G,H). Accordingly, miR-1a-3p can directly target Map3k1 to regulate the MAPK pathway, inhibiting apoptosis of LS8 at high fluoride concentration (Fig. 6).

**Table 3:** Differential target genes enriched by miR-1a-3p in MAPK signal pathway

Gene	Position in the UTR	Seed match
*Map3k1*	1593-1599	7mer-m8
*Bdnf*	213-219	7mer-m8
*Rasa1*	118-125	8mer
*Igf1*	186-193	8mer
*Nfatc3*	2232-2238	7mer-m8
*Atf2*	1326-1333	8mer
*Met*	537-543	7mer-m8
*Rap1b*	66-72	7mer-1A
*Braf*	6565-6571	7mer-m8

Position in the UTR indicates the binding site of miRNA and target gene seed match: 8mer > 7mer-m8 > 7mer-1A > 6mer

## Discussion

At present, the mechanism of dental fluorosis is complicated, and several investigations are underway. The transcriptome alterations in LS8 cells before and after fluoride exposure have not been reported. This study analyzed the effects of high fluoride on miRNA expression of LS8 cells using miRNA-seq. miR-1a-3p was significantly down-regulated in fluoride-exposed LS8, and its target genes were significantly enriched the MAPK signal pathway, which was verified by RT-qPCR. miR-1-3p can reduce the expression of SFRP1, thus promoting bone formation and mineral density and preventing osteoporosis [13]. Nakamura et al. found that the expression of miRNA-1 in mouse tooth germs lasted until 16.5 days and gradually decreased on the 1st and 3rd day after birth, reducing the proliferation rate of tooth epithelial cells. This indicates that miR-1 is essential to tooth germ development [14]. *In vitro*, miR-1a-3p promoted the proliferation and inhibited apoptosis of fluoride-stained LS8.

Extracellular signal-regulated kinase (ERK), p38, and c-jun N-terminal kinases are highly conserved serine-threonine protein kinases in the MAPK family. ERK1/2, downstream of p38MAPK, is composed of ERK1 and ERK2, which perform the same function [15]. The formation of tooth hard tissue and tooth morphology depends on the P38MAPK signal pathway, while the ERK signal pathway regulates tooth development and tooth enamel production [16]. The phosphorylated MAPK family inhibits apoptosis by increasing Bcl-2, an anti-apoptotic protein, and decreasing Bax, a pro-apoptotic protein. Under non-phosphorylation, Bax can form a heterodimer with Bcl-2 named Bax/Bcl-2, which makes Bcl-2 lose its function of supporting the mitochondrial outer membrane integrity, inducing cell apoptosis [5]. However, excessive fluoride can inhibit enamel mineralization by decreasing the phosphorylation of p38MAPK and ERK1/2 in LS8 [7]. On the contrary, inhibiting the JNK signal pathway can reduce NaF-induced LS8 cell apoptosis [17]. The results showed that fluoride exposure increased p38MAPK and ERK1/2 expression in LS8. The expression of phosphorylated p-p38MAPK and p-ERK1/2 decreased. Furthermore, the expression of anti-apoptotic protein Bcl-2 decreased, and the expression of pro-apoptotic protein Bax increased, indicating that excessive fluoride might increase the expression of MAPK signaling proteins p38MAPK and ERK1/2, reduce the expression of p-p38MAPK and p-ERK1/2, weaken phosphorylation and apoptosis inhibition, and promote ameloblast apoptosis, which is highly consistent with the above-mentioned results. However, the overexpression of miR-1a-3p reversed this process.

MiR-1a-3p can inhibit apoptosis and promote the proliferation of fluoride-stained LS8 cells by regulating the MAPK signal pathway. So, can we find the target genes that miR-1a-3p directly regulates the MAPK pathway? Considering the possibility of false positive of target genes predicted by bioinformatics, we analyzed the gene sequence of miR-1a-3p and used several databases to screen the target genes of miR-1a-3p, including Targetscan, miRWalk, and miRDB. The results showed that most of the target genes of miR-1a-3p were not confirmed. It was found that there were binding sites between miR-1a-3p and 3'UTR of *Map3k1*, and Map3k1 protein was located at the key node of the MAPK signal pathway. The complementarity between miRNA targets and seed regions, the conservation of miRNA target sites across species, and the thermal stability of miRNA-mRNA double strands are used in computer software target prediction [18]. There is a 7-nt matching region between the target gene and miRNA; therefore, the seed region of microRNA is the 2nd–8th (7mer-m8) or the 2nd–7th (7mer-A1) nucleotides of miRNA [18]. The 7mer-m8 region of Map3k13'UTR, which is located in the seed region, is where miR-1a-3p and Map3k1 connect. The sequence and function of the miR-1 family are very similar across species, and *Map3k1* 3'UTR is very reliable across species. The complementary free energy of miRNA/mRNA is in thermodynamic stability. When microRNA binds to mRNA, after which it has a higher affinity, the subsequent double strands have reduced free energy. To meet the above-described requirements, consideration of the binding site is necessary. The binding site must be at least 15 nt away from the base of the termination codon [19], and the binding site of miR-1a-3p and *Map3k1* target gene is at 3'UTR 1593–1599 bp, meeting the above-mentioned requirements.

This study showed that excessive fluoride promoted apoptosis of LS8 by promoting Map3k1 expression. Therefore, miRNAs can affect cell proliferation by interacting with Map3k1. Additionally, the overexpression of miR-1a-3p increased the expressions of Map3k1 mRNA and protein. Based on double luciferase assay, miR-1a-3p directly negatively regulated the expression of Map3k1. On the other hand, ERK1/2 is downstream of p38MAPK, whereas Map3k1 protein is upstream. In this study, fluoride-induced LS8 down-regulated the expression of miR-1a-3p, which directly targeted *Map3k1*, activated downstream signal pathways p38MAPK and ERK1/2, and decreased apoptosis and proliferation of LS8, while the overexpression of miR-1a-3p reversed this process.

This discovery not only reveals the new mechanism of post-translational gene regulation of Map3k1 but also broadens the biological function of miR-1a-3p by directly negatively regulating the MAPK signal pathway to Map3k1, which is important in the apoptosis of LS8 induced by excessive fluoride. However, the specific molecular mechanism of how target gene *Map3k1* of miR-1a-3p affects apoptosis of fluoride-exposed LS8 needs further investigation. However, there are still some shortcomings in this study, for example, whether the change of the expression of *Map3k1* can cause the change of miR-1a-3p expression? And whether the change of the expression of *Map3k1* might change the regulation of miR-1a-3p on fluoride-stained LS8 cells has not been further studied. Furthermore, the effect of miR-1a-3p and its target gene *Map3k1* on dental fluorosis needs to be further verified in animal experiments.

## Conclusion

MiR-1a-3p might inhibit apoptosis and promote the proliferation of fluoride-exposed LS8. The mechanism might be related to the direct targeting of miR-1a-3p to inhibit Map3k1, which might inhibit the activity of the MAPK signal pathway and promote its phosphorylation.
